# Disease severity determines health-seeking behaviour amongst individuals with influenza-like illness in an internet-based cohort

**DOI:** 10.1186/s12879-017-2337-5

**Published:** 2017-03-31

**Authors:** Maria Peppa, W. John Edmunds, Sebastian Funk

**Affiliations:** London School of Hygiene and Tropical Medicine, Keppel Street, London, WC1E 7HT UK

**Keywords:** Influenza, influenza-like illness, influenza surveillance, healthcare-seeking behaviour, health services

## Abstract

**Background:**

Seasonal influenza epidemics place considerable strain on health services. Robust systems of surveillance are therefore required to ensure preparedness. Sentinel surveillance does not accurately capture the community burden of epidemics as it misses cases that do not present to health services. In this study, Flusurvey (an internet-based community surveillance tool) was used to examine how severity of disease influences health-seeking behaviour in the UK.

**Methods:**

Logistic regression with random effects was used to investigate the association between health-seeking and symptom severity, duration of illness and reduction in self-reported health-score over four flu seasons between 2011 and 2015.

**Results:**

The majority of individuals did not seek care. In general, there was very strong evidence for an association between all severity indicators and visiting a health service (*p* < 0.0001). Being female (OR 1.62, 95% CI 1.23–2.14, *p* = 0.0003) and a self-diagnosis of the flu (OR 3.39, 95% CI 2.38–4.83, *p* < 0.0001) were also associated with increased likelihood of visiting a health service. During the 2012–13 and 2014–15 flu seasons, there was a significantly larger proportion of individuals with more severe sets of symptoms and a longer duration of illness. Despite this, the proportion of individuals with particular sets of symptoms visiting a health service showed only very slight variation across years.

**Conclusions:**

Traditional surveillance systems capture only the more severe episodes of illness. However, in spite of variation in flu activity, the proportion of individuals visiting a health service remains relatively stable within specific sets of symptoms across years. These data could be used in combination with data on consultation rates to provide better estimates of community burden.

**Electronic supplementary material:**

The online version of this article (doi:10.1186/s12879-017-2337-5) contains supplementary material, which is available to authorized users.

## Background

Seasonal influenza epidemics are estimated to be annually responsible for up to five million severe cases of illness and up to half a million deaths worldwide [1]. Influenza varies in its clinical manifestations; whilst some infections are asymptomatic, others lead to hospital admissions and death [1]. Furthermore, circulating viral strains are able to influence seasonal severity and hospitalization rates, with rates generally being elevated in seasons dominated by H3N2 strains [2, 3]. It is clear, therefore, that influenza warrants robust systems of surveillance to ensure preparedness, prevention and control.

Influenza surveillance in the UK primarily relies on reports of consultations for influenza-like illness (ILI) from primary care physicians. To supplement this, ‘Flusurvey’ was launched in 2009 as part of ‘Influenzanet’. In its current form, Influenzanet is a network of 10 countries, aiming to monitor ILI rates across Europe in a consistent manner through the use of online questionnaires [4]. Flusurvey is open to all UK residents and allows participants to report the presence or absence of specific symptoms, their health-seeking behaviour and a range of demographic, medical and other behavioural data.

Flusurvey reproduces trends in ILI incidence captured by traditional surveillance, particularly during seasonal epidemics [5]. Demographic and medical history information collected from participants has been used to show that unvaccinated individuals, those with underlying health conditions and those with contact with children are more likely to report symptoms consistent with ILI [6]. Behavioural information has been used to show that the reduction in contacts children make outside of school terms can explain a decrease in ILI incidence during these periods [7]. Data collected on illness duration and the participant’s perceived health-score have allowed for the calculation of quality-adjusted life-days lost for reported episodes of illness and have shown that ILI episodes are associated with a greater loss than acute respiratory infections (ARIs) [8]. Finally, data on vaccination status collected in Flusurvey have also been used for rapid evaluation of vaccine efficacy [9, 10].

Although traditional surveillance systems continue to be useful, a frequently cited limitation is that they rely on patients seeking medical attention and fail to capture those with symptoms who do not seek care. There are data to suggest that the majority of adults with flu-related symptoms did not seek healthcare during the 2009 pandemic [11, 12]. Behaviour during pandemics is not necessarily an indicator of behaviour during seasonal epidemics. However, if low proportions of individuals with ILI seek care during seasonal epidemics and if those who do are more likely to have more severe illness, this implies that traditional surveillance can inflate severity estimates [13]. In this paper we use Flusurvey data to define severity indicators for ILI and use these indicators to assess how the severity of illness is associated with the odds of visiting a health service. We also identify other factors strongly associated with health-seeking behaviour, irrespective of disease severity.

## Methods

All analyses were carried out with STATA/IC 14. In the UK, there were 3 waves of the 2009 H1N1 pandemic - including the 2010–2011 season. Due to increased media coverage and public awareness of flu over these seasons, this study utilized Flusurvey data collected from UK residents during 2011–12, 2012–13, 2013–14 and 2014–15. Only reports in which individuals had confirmed the presence of at least one symptom were used. To ensure that the whole influenza season was captured each year but that episodes outside this were not included, only records with symptom start dates occurring between September 1st and May 31st were retained. The use of Flusurvey for the purposes of this research was approved by the London School of Hygiene and Tropical Medicine Ethics Committee (reference number 9800).

### The Flusurvey questionnaires

Participants completed an initial questionnaire on their demographic and background medical information at the beginning of each season which they were able to update if necessary. Weekly e-mail reminders to complete symptom questionnaires were then sent out. Participants reporting symptoms of ILI were prompted to fill in information relating to their illness such as the start and end date of symptoms, whether they’d sought care, and their opinion about what caused their illness (self-diagnosis). Moreover, participants could indicate whether symptoms reported in consecutive surveys where due to the same bout of illness. We combined subsequent reports from the same bout of illness into a disease episode with a given symptom onset and end date. From 2012 onwards, participants were asked to state their perceived health-score every week, independently of whether they recorded symptoms or not (where 100 was perfect health and zero was death). The health-score measurement scale was adapted from the widely used EQ-5D ‘Visual Analogue Scale’, in which participants are asked to score their current health state on a scale ranging from 0 “worst imaginable” to 100 “best imaginable”. The principle adaptation for ease of online data collection being that participants are asked to enter the value in a box rather than indicating it as a line on a “thermometer” [14]. The EQ-5D ‘Visual Analogue Scale’ has been used to examine flu-related decline in quality of life and results have been shown to be similar when using the EQ-5D questionnaire which assesses quality of life through questions on mobility, self-care, usual activities, pain/discomfort and anxiety/depression [15].

### Health-care seeking behaviour

Our primary outcome of interest was visiting a health service, although making contact with a health service (via the internet or telephone) was also examined. Health-seeking behaviour information was recorded by asking what type of service a patient had visited (i.e. face-to-face) and/or contacted (i.e. internet or telephone). In both instances, patients were given the option of selecting “No” if no visit or contact of any kind took place. Not selecting “No” automatically coded individuals as having visited or contacted a health service of some kind, thus preventing double counting in case individuals had visited or contacted more than one service [12].

### Severity indicators

Symptom severity, illness duration and health-score decrease were chosen as potential illness severity indicators. Using clinical case definitions from the European Centre for Disease Prevention and Control (ECDC), episodes of ILI and acute respiratory illness (ARI) were first distinguished. The ECDC defines ARI as a sudden onset of symptoms in combination with at least one of four respiratory symptoms (cough, sore throat, shortness of breath, coryza) [16]. ILI is defined by a sudden onset of symptoms, at least one of three respiratory symptoms (cough, sore throat, shortness of breath) and at least one of four systemic symptoms (fever, malaise, headache, myalgia) [16].

Based on this definition, an individual need not have a fever in order to have ILI. However, because other ILI case definitions require the presence of a fever and because individuals with a fever are more likely to have more severe illness and/or perceive their illness to be more severe, we decided to have separate ILI subcategories to reflect the absence (ILI_No Fever_) or presence (ILI_Fever_) of a fever.

Flu can sometimes lead to bacterial chest infections such as bronchitis or pneumonia and such infections can involve the production of phlegm. However, these conditions may also be the result of other viral or bacterial infections [17]. Individuals who fulfilled ILI criteria, including the presence of a fever, but who also had phlegm as an additional symptom were categorized separately (ILI_Fever_ with phlegm). This was done in order to account for the fact that these individuals may have more severe ILI (and/or may perceive their disease to be more severe) but also to highlight that individuals with these symptoms may be more likely to have other causes of disease.

Individuals only had to present with one respiratory symptom to fulfil the ARI definition whereas they had to present with at least one systemic and one respiratory symptom to fulfil the ILI definition. Those with ILI_Fever_ with phlegm had to fulfil ILI criteria (including the presence of fever) but had to have phlegm as an additional symptom. We therefore used ARI episodes as a baseline and symptom severity was ordered as follows: ARI < ILI_No fever_ < ILI_Fever_ < ILI_Fever_ with phlegm. Categories were non-overlapping, such that individuals could only be in one of them.

The duration of an episode in days was calculated by subtracting the start date of symptoms from the end date of symptoms. For the 2012–13, 2013–14 and 2014–15 seasons, during which we had collected data on participants’ perceived health-score, we calculated a median health-score for each individual based on the health-score they reported when they had no symptoms. This was used with the minimum health-score reported during each episode of illness to calculate the percentage decrease in health-score from the baseline in order to account for the variability in the way individuals conceptualize health-score. To examine the association between the severity indicators, Χ^2^ tests were performed.

### Data cleaning and preparation

Start and end dates of symptoms were marked as erroneous if they were more than 60 days apart or if the end date occurred before the start date. While most ILI episodes resolve within a few days, the 60-day limit was chosen because high-risk individuals might report prolonged symptoms, particularly if they developed further complications such as bronchitis or pneumonia. Where it was clear that participants had incorrectly entered one of the dates and this had caused symptom duration to exceed 60 days, the date and duration were corrected. For example, if a participant of the 2012–13 survey had entered a start date as 07 December 2002 and an end date as 15 December 2012, their start date was amended to 07 December 2012. If dates were the wrong way around, they were switched. Where possible, the start date of a fever was used to gauge whether an amendment was reasonable. If dates were far apart and no obvious error was found or it was unclear how to correct the dates but the same individual had submitted previous records correctly, dates and duration were converted to missing values. If this was the case but there were no other records from the same individual, the record was excluded.

Age was calculated by subtracting birthdates from symptom end dates. If individuals were older than 100 but had successfully completed multiple records, their age was converted to ‘missing’ as it was considered likely that their birthdate was an error. For reasons of reliability, records for those under the age of ten were only left unchanged if it was indicated that the surveys had been filled out by another household member. If this was not the case but the individual had indicated they were authentic by completing multiple records successfully, birthdate and age were considered to be mistakes and converted to ‘missing’. Otherwise, records were excluded. For education, the highest qualification attained was used where an individual indicated they were still in education but had previous qualifications. Individuals who only selected “still in” education and had no previous qualifications were defined as having no qualifications at the time of submission. Finally, health-score and baseline health-score were converted to missing values if they fell outside the range of 0–100.

Only participants who had reported “no symptoms” at least once were included in the analysis, to exclude those with chronic symptoms, or those who only registered to report a bout of ILI.

### Descriptive analysis

Descriptive analyses of demographic and medical background data were carried out in each season individually and in the combined dataset. Because most individuals had submitted multiple reports, datasets were reduced so that only the most up to date report by each individual was kept and used for this. Descriptive analyses of episodes of illness were carried out in the full databases (containing all records for each individual) for each season and combined.

### Statistical analysis

Logistic regression with random effects to adjust for clustering by individual was used to investigate the association between severity indicators and health-seeking behaviour. Confounders considered for inclusion in the model were: gender, age, smoking status, flu vaccination status, all underlying health conditions, highest educational qualification (as a proxy for socioeconomic status), main type of transport used, length of time spent on transport, the presence of children in the household, the participant’s own thoughts on the cause of their illness (self-diagnosis) and season. In addition to these variables, we also adjusted for whether the individual was ill during the period in which influenza virus was circulating. The timing and duration of influenza circulation differs each year and influenza circulation was defined as the period in which ≥5% of specimens sent for laboratory testing were positive for influenza virus, according to the Respiratory DataMart System [18–21]. Although there were other variables of interest, such as pregnancy and occupation, these were not explored due to high levels of missing data and low numbers of observations within strata.

Univariate analysis of all variables was first carried out. Multivariate analysis was then performed which included all the severity indicators and used a change-in-estimate approach to select confounders for inclusion in the final model (Appendix 1). As 2011–12 contained no data on health-score, models looking at all three severity indicators were limited to data from 2012 onwards. However, all models that did not include health-score were run with and without 2011–12 and compared.

Season was controlled for in the full models in order to describe the general association between severity of illness and health-seeking behaviour. However, because different influenza strains between years could potentially alter symptom severity and disease duration, we also tested for effect modification between season and these severity indicators. It was thought that the health-score reported by an individual would be determined, in part, by the severity of their symptoms and the duration of their disease. Therefore, interaction of season with percentage decrease in health-score was not examined because it was thought that if seasonal effect modification occurred, it would be apparent at the biological levels of symptoms and duration.

## Results

### Participant demographics and medical histories

The total number of participants and the number of individuals that reported the presence of at least one symptom varied between years. During the 2011–12 flu season, 1669 individuals reported having any symptom over the course of the season (out of 2201, 76%) whereas 3443 (out of 4248, 81%), 3292 (out of 4559, 72%) and 3301 (out of 4202, 79%) individuals reported symptoms during 2012–13, 2013–14 and 2014–15, respectively. Despite this, demographics and medical histories of individuals across flu seasons were broadly similar (Table 1). In the combined dataset, 63.7% (7459/11,705) of individuals were female. There were fewer individuals at the age group extremes; 6.6% (774/11,705) of individuals were 0–18 years old and 11.9% (1395/11,705) were over 66 years old. The majority of individuals had attained higher education qualifications whilst only 5.1% (601/11,705) reported having none. Asthma, allergies, heart disease and diabetes were the most common underlying health conditions.

**Table 1:** Characteristics of study participants. Values given in per cent, with number of individuals in parentheses

	Percentage of individuals per category in each year (No.)				
	2011–12 (*N* = 1669)	2012–13 (*N* = 3443)	2013–14 (*N* = 3292)	2014–15 (*N* = 3301)	Combined (*N* = 11,705)
Gender					
Male	39 (651)	37.4 (1287)	35.6 (1173)	34.4 (1135)	36.3 (4246)
Female	61 (1018)	62.6 (2156)	64.4 (2119)	65.6(2166)	63.7 (7459)
Age (years)					
0–18	4.9 (81)	5.2 (179)	9.5 (314)	6.1 (200)	6.6 (774)
19–45	46.6 (777)	44 (1515)	39.9 (1313)	37.5 (1237)	41.4 (4842)
46–65	37.2 (620)	38.8 (1336)	37.2 (1224)	41.4 (1367)	38.9 (4547)
≥ 66	10.3 (172)	10.8 (372)	12 (396)	13.8 (455)	11.9 (1395)
Highest qualification achieved					
None	2.9 (49)	2 (69)	11.9 (392)	2.8 (91)	5.1 (601)
GCSEs/equivalent	5.9 (98)	8.5 (294)	7 (230)	7.8 (257)	7.5 (879)
A-Levels/equivalent	12.2 (203)	15.5 (534)	11.9 (390)	13.2 (436)	13.4 (1563)
Undergraduate degree	25.4 (424)	25.5 (879)	24.4 (803)	24.9 (822)	25 (2928)
Post-graduate degree	50.1 (836)	41 (1413)	40.6 (1336)	42.2 (1392)	42.5 (4977)
Transport used					
Walk/Bike	27.9 (466)	21.5 (741)	22.4 (737)	20.7 (684)	22.5 (2628)
Personal transport^a^	43.1 (719)	51.6 (1778)	50.9 (1674)	53.9 (1780)	50.8 (5951)
Public transport	28.2 (470)	26.5 (913)	26.3 (866)	25 (824)	26.3 (3073)
Other	0.8 (14)	0.3 (11)	0.5 (15)	0.4 (13)	0.5 (53)
Risk factor					
Children in household	28.5 (476)	32.4 (1114)	33.2 (1092)	31.5 (1038)	31.8 (3720)
Smokers	9.3 (155)	9.3 (320)	7 (230)	6.8 (225)	8 (930)
Vaccinated for Flu	40 (667)	34.5 (1188)	36.1 (1189)	38.4 (1266)	36.8 (4310)
Asthma	10.2 (170)	10.8 (371)	10.1 (332)	10.3 (340)	10.4 (1213)
Allergies	37.6 (628)	36.5 (1257)	38.9 (1279)	39 (1289)	38 (4453)
Diabetes	2.8 (47)	3.6 (123)	2.8 (91)	3 (98)	3.1 (359)
Chronic Lung Disease	0.9 (15)	1.1 (38)	1 (34)	0.9 (30)	1 (117)
Heart Disease	3.8 (64)	3.8 (130)	4.2 (138)	3.9 (129)	3.9 (461)
Renal Disease	0.4 (6)	0.3 (11)	0.2 (6)	0.4 (13)	0.3 (36)
Immunodeficiency	1 (17)	1.7 (59)	1.2 (39)	1.8 (59)	1.5 (174)

^a^e.g. car or motorbike

### Number of episodes contributed by individuals and missing data

In total, there were 23,961 reported episodes containing at least one symptom in the combined dataset. 42.9% (5021/11,705) of individuals contributed just one such episode, 29% (3393/11,705) contributed two and 15.7% (1832/11,705) contributed three. Remaining individuals contributed between four to nine episodes each. Out of these 23,961 episodes, 9470 (39.5%) could be classified as being an ARI or ILI whilst the rest were classified as “other” and were not examined.

Data cleaning to remove implausible values resulted in a small proportion of missing data for age (1.33%; 126/9470), duration of illness (0.08%, 8/9470) and whether or not the individual was ill during the period of time in which influenza was circulating (0.08%; 8/9470). There were also missing data for educational qualifications achieved (6.34%; 600/9470), smoking (0.08%; 8/9470), influenza vaccination (0.14%; 13/9470) and self-diagnosis (0.23%; 22/9470). Because no health-score data were collected for 2011–12, 28.5% (2696/9470) of observations were missing for percentage decrease in health-score. Missing data in these variables were not associated with the outcomes of interest.

### Description of flu seasons, severity indicators and health-seeking behaviour

The proportion of all symptom reports in Flusurvey that could be classified as ARI or ILI cases varied by flu season. In 2011–12, 37.7% (1302/3457) of disease episodes were defined as ARI or ILI cases. A similar proportion of 36.9% (2544/6902) was seen in 2013–14. Flu seasons in 2012–13 and 2014–15 presented a slightly greater proportion of ARI and ILI episodes, 41.4% (2947/7118) and 41.3% (2677/6484) respectively. In the combined dataset, 39.5% (9470/23,961) of disease episodes could be classified as being ARI or ILI.

For all seasons, as the number of symptoms and duration of illness increased, the proportion of episodes in those categories decreased (Table 2). A clear trend was not observed for percentage decrease in health-score. All pairings of severity indicators showed strong evidence of being correlated with one another (Χ^2^ *p* < 0.001) (Additional file 1: Figure S1).

**Table 2:** Distribution of severity indicators by flu season. Values given in per cent, with number of illness episodes in parentheses

	Percentage of individuals per category in each year (No.)				
	2011–12	2012–13	2013–14	2014–15	Combined
Symptoms					
ARI	45 (586/1302)	32.3 (951/2947)	41.2 (1049/2544)	33.1 (887/2677)	36.7 (3473/9470)
ILI–No fever	33.2 (432/1302)	31.4 (925/2947)	36.4 (926/2544)	32.2 (863/2677)	33.2 (3146/9470)
ILI-Fever	13.4 (175/1302)	21.1 (623/2947)	13.8 (352/2544)	21.1 (564/2677)	18.1 (1714/9470)
ILI-Fever, with phlegm	8.4 (109/1302)	15.2 (448/2947)	8.5 (217/2544)	13.6 (363/2677)	12 (1137/9470)
Duration (days)					
0–3	56.2 (732/1302)	50.9 (1501/2947)	56.9 (1443/2537)	51 (1364/2676)	53.3 (5040/9462)
4–7	26.8 (349/1302)	28.3 (834/2947)	24.5 (622/2537)	28.1 (752/2676)	27 (2557/9462)
8–14	11.8 (154/1302)	13 (384/2947)	12.7 (322/2537)	12.8 (343/2676)	12.7 (1203/9462)
≥ 15	5.2 (67/1302)	7.7 (228/2947)	5.9 (150/2537)	8.1 (217/2676)	7 (662/9462)
Health-score decrease					
0–10%	-	21.5 (463/2153)	26.3 (573/2181)	22 (536/2440)	23.2 (1572/6774)
10.1-%	-	21.7 (468/2153)	24.5 (535/2181)	25.3 (618/2440)	23.9 (1621/6774)
20.1–30%	-	16.1 (347/2153)	17.8 (388/2181)	15.7 (382/2440)	16.5 (1117/6774)
30.1–50%	-	22.4 (483/2153)	21.2 (463/2181)	21.6 (527/2440)	21.7 (1473/6774)
≥ 50.1%	-	18.2 (392/2153)	10.2 (222/2181)	15.5 (377/2440)	14.6 (991/6774)

2012–13 and 2014–15 had a significantly smaller proportion of ARI episodes but a significantly larger proportion of ILI_Fever_ and ILI_Fever_ with phlegm than 2011–12 and 2013–14 (*p* = 0.0001) (Table 2). Furthermore, the proportion of individuals with the shortest duration of illness (0–3 days) was significantly greater in 2011–12 and 2013–14 than in 2012–13 and 2014–15 whereas the converse was true for the longest duration of illness (≥15 days) (*p* = 0.0001) (Table 2).

**Fig. 1:**
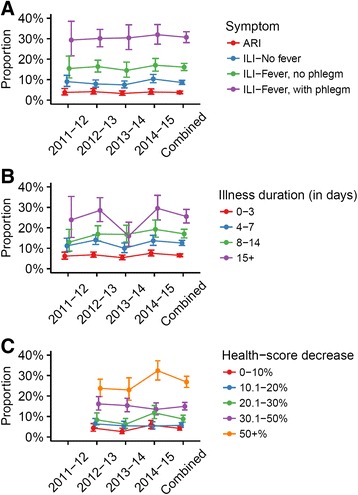
Proportion of illness episodes resulting in a healthcare service visit, by severity indicator

The majority of patients did not visit a health service. However, a greater proportion of individuals with more severe symptoms visited a health service than with less severe symptoms (Fig. 1, Additional file 2: Table S1A). Similar trends were seen in those with longer illness durations and greater health-score decreases (Fig. 1, Additional file 2: Table S1A). Although health-seeking behaviour was generally lower in 2011–12 and 2013–14, the differences between seasons were not considerable, although in general the proportion of visits appeared more stable across years in individual symptom severity categories than in duration and health-score decrease categories (Fig. 1, Additional file 2: Table S1A). Results were similar for contacting (but not visiting) a health service, although overall a greater proportion of individuals visited rather than contacted a service (Additional file 3: Table S1B).

Finally, epidemic curves for each symptom group and for each season were compared to ILI consultation rates from Public Health England (PHE) (Additional file 4: Figure S2). Trends in the epidemic curves of ILI_Fever_ with phlegm in particular appeared to reflect the trends in consultation rates. This suggested that certain groups of symptoms could potentially act as good predictors of health-seeking behaviour.

### Symptom severity, illness duration and health-score were associated with health-seeking behaviour

There was very strong evidence for an association between all three severity indicators and visiting a health service in both univariate and fully-adjusted models (*p* < 0.0001) (Table 3). The odds of health-seeking behaviour increased as symptom severity increased, with the odds of visiting a health service being highest in those with ILI_Fever_ with phlegm Their odds were 5.99 (95% CI 3.75–9.56) times higher than those with ARI (Table 3). The odds of visiting a health service increased as illness duration increased. Those ill for over two weeks had the highest odds of visiting a health service, they were 4.62 (95% CI 3.03–7.03) times more likely to visit a health service compared to those who were ill for 0–3 days (Table 3). Finally, those with a reported decrease in health-score above 20% also showed increased odds of visiting a health service. Those with a decrease ≥50.1% had the highest odds of visiting a health service, they were 5.54 (95% CI 3.41–8.99) times more likely to visit than those with a 0–10% decrease (Table 3). Finally, the full model showed strong evidence for clustering by individual (ρ = 0.38, *p* < 0.001). Results for contacting a health service were similar (Additional file 5: Table S2).

**Table 3:** Crude^a^ and adjusted^b^ ORs for visiting a health service, by severity indicator

Severity Indicator	Crude ORs (95% CI)^a^	Fully-adjusted ORs (95% CI)	Likelihood Ratio Test *P*-value for fully-adjusted model
Symptoms			
ARI	1	1	<0.0001
ILI-No Fever	2.72 (2.12–3.48)	1.58 (1.11–2.25)	
ILI-Fever	6.34 (4.82–8.35)	2.52 (1.67–3.80)	
ILI-Fever, with phlegm	17.82 (12.95–24.51)	5.99 (3.75–9.56)	
Duration (days)			
0–3	1	1	<0.0001
4–7	2.35 (1.92–2.86)	2.18 (1.63–2.91)	
8–14	3.59 (2.82–4.56)	4.03 (2.81–5.79)	
≥ 15	6.99 (5.22–9.36)	4.62 (3.03–7.03)	
Health-score decrease (%)			
0–10	1	1	<0.0001
10.1–20	1.46 (1.00–2.11)	1.56 (1.00–2.43)	
20.1–30	2.58 (1.76–3.79)	2.49 (1.56–3.97)	
30.1–50	5.32 (3.70–7.65)	3.73 (2.39–5.81)	
≥ 50.1	14.38 (9.45–21.88)	5.54 (3.41–8.99)	

^a^The crude ORs include all four years of data. Due to the inclusion of health-score, for which data was not collected in the 2011–12 season, the fully-adjusted model excludes 2011–12. Crude analyses excluding 2011–12 showed similar results to the crude analyses presented here (Additional file 6: Table S3). ^b^Adjusted for all other severity indicators and confounders

### Association between confounders and health-seeking behaviour

After adjusting for all variables, being female (OR 1.62, 95% CI 1.23–2.14, *p* = 0.0003), being diabetic (OR 2.78, 95% CI 1.55–5.00, *p* = 0.0007), having asthma (OR 1.56, 95% CI 1.08–2.25, *p* = 0.0167) and a self-diagnosis of flu (OR 3.39, 95% CI 2.38–4.83, *p* < 0.0001) showed evidence for an association with visiting a health service (Table 4). There was also some evidence to suggest age was associated with visiting a health service (*p* = 0.0387). Individuals who were between 19 and 45 years old were less likely to visit a health service than those under 18 years old (OR 0.40, 95% CI 0.18–0.88). Older adults did not appear to be more or less likely to visit a health service than children as their confidence intervals crossed one. Following adjustment, only having chronic lung disease (OR 2.34, 95% CI 1.10–4.99, *p* = 0.0348) and a self-diagnosis of flu (OR 3.43 95% CI 2.44–4.83, *p* < 0.0001) were associated with contacting a health service (Additional file 5: Table S2).

**Table 4:** Crude^a^ and adjusted^b^ ORs for visiting a health service, by other variables of interest

Variable	Crude ORs (95% CI)^a^	Fully-adjusted ORs (95% CI)	Likelihood Ratio Test *p*-value for fully-adjusted model
Influenza circulating			
No	1	1	0.1107
Yes	1.07 (0.90–1.27)	0.79 (0.59–1.05)	
Gender			
Male	1	1	0.0003
Female	1.46 (1.22–1.74)	1.62 (1.23–2.14)	
Age (years)			
0–18	1	1	0.0387
19–45	0.48 (0.35–0.67)	0.40 (0.18–0.88)	
46–65	0.66 (0.48–0.91)	0.49 (0.22–1.08)	
≥ 66	0.66 (0.45–0.97)	0.63 (0.27–1.47)	
Highest qualification			
None	1	1	0.1996
GCSEs/equivalent	1.03 (0.66–1.60)	0.93 (0.47–1.85)	
A-Levels/equivalent	0.95 (0.63–1.43)	0.86 (0.44–1.67)	
Undergraduate	0.63 (0.42–0.93)	0.62 (0.32–1.21)	
Post-graduate	0.60 (0.41–0.87)	0.69 (0.36–1.34)	
Transport used			
Walk/Bike	1	1	0.1105
Personal transport	1.54 (1.23–1.92)	1.31 (0.94–1.83)	
Public transport	0.99 (0.76–1.28)	0.93 (0.63–1.38)	
Other	4.95 (1.63–15.00)	1.76 (0.20–15.33)	
Children in household			
No	1	1	0.4407
Yes	1.19 (1.00–1.42)	1.11 (0.85–1.46)	
Smoking status			
No	1	1	0.0524
Yes	1.44 (1.10–1.90)	1.47 (1.00–2.17)	
Flu vaccine			
No	1	1	0.0895
Yes	1.09 (0.92–1.29)	1.27 (0.96–1.68)	
Asthma			
No	1	1	0.0167
Yes	1.86 (1.47–2.36)	1.56 (1.08–2.25)	
Allergies			
No	1	1	0.3267
Yes	1.21 (1.02–1.42)	1.13 (0.88–1.46)	
Diabetes			
No	1	1	0.0007
Yes	2.17 (1.46–3.22)	2.78 (1.55–5.00)	
Chronic Lung Disease			
No	1	1	0.1912
Yes	2.78 (1.53–5.03)	1.72 (0.77–3.84)	
Heart Disease			
No	1	1	0.2235
Yes	1.53 (1.05–2.24)	1.42 (0.81–2.47)	
Renal Disease			
No	1	1	0.2154
Yes	1.61 (0.51–5.09)	0.31 (0.05–2.14)	
Immunodeficiency			
No	1	1	0.3764
Yes	2.57 (1.48–4.47)	1.44 (0.65–3.19)	
Self-diagnosis			
Cold	1	1	<0.0001
Flu	8.19 (6.70–9.99)	3.39 (2.38–4.83)	
Flu Season (year)			
2011–12	0.72 (0.55–0.94)	-	0.0621
2012–13	1	1	
2013–14	0.65 (0.52–0.82)	0.87 (0.63–1.20)	
2014–15	1.09 (0.89–1.34)	1.25 (0.95–1.64)	

^a^The crude ORs include all four years of data. Due to the inclusion of health-score, for which data was not collected in the 2011–12 season, the fully-adjusted model excludes 2011–12. Crude analyses excluding 2011–12 showed similar results to the crude analyses presented here (Additional file 6: Table S3). ^b^Adjusted for all other variables and severity indicators

### Health-seeking behaviour by season and by flu circulation

Univariate models revealed very strong evidence for an association between flu season and visiting a health service (*p* < 0.0001), with the odds of health-seeking behaviour being lowest in 2011–12 and 2013–14 (Table 4). However, the upper ends of confidence intervals were close to one in this model. Evidence for an association in the fully-adjusted model was considerably weaker (*p* = 0.0621), with confidence intervals crossing one (Table 4). Because the severity of flu seasons may vary, it was thought that flu season could interact with and modulate symptom severity and/or duration of illness. There was no evidence, however, for effect modification between season and symptom severity (*p* = 0.30). There was also no evidence for effect modification between season and illness duration in the visit model (*p* = 0.67). Corresponding ORs did vary by individual year but there was considerable overlap between confidence intervals within years, preventing further conclusions from being drawn (Additional file 7: Table S4A and Additional file 8: Table S4B).

Finally, there was no evidence to suggest that individuals who were ill during the period of time in which influenza virus was circulating in the population were more likely to visit or contact a health service compared to those who were ill outside of this, between September and May (Table 4, Additional file 5: Table S2).

## Discussion

### Limitations

The main strength of Flusurvey, relative to traditional surveillance, lies in its ability to capture information about illness from those who may not seek care. The caveat, however, is that those who volunteer to participate may not be representative of the UK population. Participants tended to be female, more highly educated and less likely to be over 65 or under 18 years of age. We included all these variables in the regression analysis to adjust for any biases, but results should be interpreted cautiously. More generally, those who register with Flusurvey may have particular levels of concern or interest with respect to ILI in a way that is not captured by the background variables but affects their healthcare-seeking behaviour, which could bias our results.

Flusurvey allows for the collection of a large range of information from its participants that may not be captured in a clinical setting. However, because the survey is self-administered, participants may on occasion make errors in data entry which may yield implausible and/or contradictory values. In addition, data on symptoms experienced, the start and end dates of symptoms and decrease in health-score all depend on the participant remembering and reporting their illness accurately. This may not always be possible and may also depend on external factors, such as the media influencing perceptions. The latter is particularly the case for health-score which is a subjective measurement and will depend on the individual’s beliefs of what good or poor health is. To reduce recall bias as well as the appearance of implausible values reported for other variables, all variables of interest were examined carefully during the data cleaning stage and reports excluded when they appeared improbable or erroneous. Additionally, the percentage decrease in health-score from each individual’s median baseline score was calculated in order to minimize the subjectivity associated with this variable. In the future, it may be useful to consider optimizing the survey such that individuals are prompted to correct any implausible entries.

It could be argued that because self-administered surveys do not involve physicians who can make a judgement on the cause of illness reported by participants, the number of cases of a disease might be inflated. We note, however, that participants are asked only to report flu-like symptoms and not symptoms related to chronic conditions and we only included those who reported “no symptoms” at least once in the past so as to exclude those mistakenly reporting such conditions. Participants are also asked to indicate what they think the cause of their illness is and our data suggested that they were generally able to gauge the severity of their illness, classifying more severe episodes as “flu-like”. Lastly, in most cases of ARI or ILI, physicians make a judgement in the absence of any laboratory evidence, and it is not clear how this would alter the number of cases compared to what we found.

Ideally, laboratory testing would have allowed ILI cases and non-cases to be recognized more robustly. However, the cost of swabbing and analysing samples precluded virological testing. Case definitions, from various public health bodies, can be used to define ILI and ARI cases in the absence of virological testing. The ECDC ILI definition is less specific than other definitions that require the presence of a fever of ≥38 °C but was used in this study because few individuals recorded their exact temperature. Although we were unable to define the temperature of the individual, we did examine subcategories of the ECDC ILI case definition by splitting episodes according to the absence or presence of a fever (ILI_No fever_ and ILI_Fever_, respectively). Furthermore, although the ECDC definition of ILI is often utilized by health professionals and surveillance systems, the ECDC cautions that other viruses as well as bacteria can cause similar symptoms to ILI and may be captured by the case definition [17]. To account for the fact that some individuals had phlegm in addition to ILI symptoms and that this might be more likely to indicate a pneumonia-like-illness rather than ILI, we also examined individuals who had ILI_Fever_ with phlegm. Overall, our results suggested that our data adequately represented influenza seasons when compared to PHE data (Additional file 4: Figure S2). A previous study has estimated that about 18% of people seroconvert (that is, become infected with influenza) over the course of a season, which is broadly in line with the rates we observed for ILI_Fever_ [22].

The large proportion of missing data and low numbers of observations within strata for some variables precluded some analyses. Pregnancy, occupation and type of care sought could not be examined for these reasons. Socioeconomic status was previously found to be associated with health-seeking behaviour during the 2009 influenza pandemic and could play a similar role during seasonal epidemics [23]. There was no good indicator of socioeconomic status available, although the highest educational qualification achieved was considered to be an adequate proxy. Despite these limitations, we were still able to examine a large number of individual risk factors where only a small proportion of entries were classified as missing and without obvious correlation with the outcome variables. In this context, it should be noted that we did not have any information on participants who may have made an attempt to complete the survey but did not finish, and we therefore cannot rule out that this may introduce a bias.

### Conclusions and implications

#### Severity of illness and service utilization

The majority of individuals with ARI or ILI did not visit a health service. This is in line with findings from Flu Watch, a cohort study collecting data on influenza from households in England, as well as findings from similar studies elsewhere which showed that the majority of individuals with ILI in other northern European countries such as Sweden and the Netherlands did not visit a doctor between 2003 and 2013 [22, 24]. Conversely, the proportion of individuals with ILI seeking care in southern European countries such as Portugal, Italy, Spain and France has been shown to be greater [24].

There was very strong evidence to suggest that more severe episodes of illness increased the likelihood of visiting a health service, even when all indicators and confounders were adjusted for. Therefore, despite the fact that all indicators were associated with each other, each severity indicator was to some extent independently associated with health-seeking behaviour. These results agreed with data from Belgium which showed that patients who sought care tended to have more symptoms and a longer duration of illness [25]. Sentinel surveillance is therefore likely to underestimate the number of influenza episodes in the community but also routinely capture more severe episodes of illness, thus distorting interpretations of seasonal severity, as previously suggested [13].

NHS guidelines recommend that otherwise-healthy individuals only seek care if symptoms have not improved after a week [26]. It was therefore expected that if individuals were following these guidelines, the odds of visiting a health service would be roughly similar for those with an illness duration of 0–3 and 4–7 days after adjusting for confounders such as age and underlying health conditions. However, those who were ill for 4–7 days were more than twice as likely to visit a service compared to those who were ill for 0–3 days. It is possible that individuals are unaware of these guidelines or are choosing to seek care earlier than recommended.

Those who fulfilled the ILI definition were more likely than those with ARI to visit a service. Furthermore, the odds of this health-seeking behaviour were seen to increase when the percentage decrease in health-score surpassed the threshold of 20.1%. Altogether, these data suggested that individuals were capable of gauging the severity of their disease and categorizing more severe episodes as “flu-like”. This is supported by an observed increase in the likelihood of visiting a health service when individuals self-diagnosed their illness as flu compared to a cold.

Although the odds of visiting a health service increased as all three severity indicators increased, there was also some overlap in confidence intervals between categories which limited further interpretation of results. This was likely due to the inclusion of confounders in the full model and subsequent sparsity of data across strata.

#### Other factors influencing service utilization

The fully-adjusted model displayed strong evidence for clustering by individual. This indicated that to some extent, visiting a health service was driven by an individual or personal component. Similar to previous studies of both seasonal and pandemic influenza, being female was associated with a greater likelihood of visiting a health service for a given level of symptom severity [11, 27]. As the presence of children in the household was adjusted for (and was not associated with visiting a health service), it was thought that the increased likelihood of women in childcare roles was not the main reason for the observed association. The decreased propensity of men to seek care has been widely reported and is considered to be the case irrespective of age and ethnicity, although it may vary according to the disease and sociocultural norms [28].

Individuals with underlying health conditions are encouraged to seek care if they suspect having influenza due to their increased risk of developing complications [26]. Previous research has shown that individuals with heart disease, asthma, lung disorders and renal disease are more likely to report illness and seek help [11, 27, 29]. This study found that having diabetes or asthma was associated with an increased likelihood of visiting a service. The low number of participants with underlying health conditions may have precluded further associations from being identified.

There was evidence to suggest that being between the ages of 19–45 was associated with decreased odds of visiting a health service. Similar observations have been made elsewhere [27]. Previously, the elderly have been found to show increased health-seeking behaviour relative to other age groups [11, 27]. Our results did not suggest increased odds of visiting a health service amongst individuals above 45 years of age. According to PHE, the impact of flu on age groups varied by season. The greatest impact was seen in young adults in 2013–14 and in the elderly in 2014–15 [18–21]. It is possible that any age-specific effects were lost when season was adjusted for or that adjusting for vaccination and underlying health conditions diluted any associations between age groups and visiting a health service. It is also possible that underrepresentation of the elderly prevented associations from being found or that the elderly and very young do indeed have similar odds of visiting a health service to each other.

Finally, our univariate model revealed evidence for an association between flu season and health-seeking behaviour, with the odds of visiting health services being lower in 2011–12 and 2013–14 than in 2012–13 and 2014–15. However, there was little support for this in the fully-adjusted model. This was not unexpected and was likely due to the fact that severity of disease was adjusted for. There was also no evidence that season modified the magnitude of the association between visiting a health service and symptom severity or duration of illness. Whilst there was strong evidence for effect modification between season and illness duration in the contact model (*p* = 0.0039) and corresponding ORs varied across years, overlap between confidence intervals within years was considerable and prevented further conclusions.

#### Seasonal severity and community burden

Our work found that amongst the four ‘respiratory disease states’ examined, their relative occurrence was as follows: ARI > ILI_No fever_ > ILI_Fever_ > ILI_Fever_ with phlegm. The large proportion of ARI and ILI_No fever_ episodes further suggests that most episodes are mild in severity and that few individuals have severe episodes, indicating that our indicators adequately captured the range in disease severity for the purposes of this research. Although ARI was the most frequently occurring type of episode when comparing all four individual disease categories, the majority of episodes that were reported included systemic symptoms and therefore fulfilled the ECDC ILI definition. Among the episodes that fulfilled the ECDC ILI definition, the majority (46–62%) tended not to have a fever. The proportion of ILI episodes in which fever was reported ranged from 24 to 32% whilst the proportion which had fever as well as phlegm was lower but still sizeable, ranging from 15 to 22%.

When examining individual years, our data suggested that there was a greater proportion of individuals fulfilling the ILI_Fever_ definition in 2012–13 and 2014–15 than in 2011–12 and 2013–14. In 2012–13 and 2014–15, approximately 21% of individuals could be defined as having ILI_Fever_, whereas 14% could be described this way in the other two seasons. This could indicate that these years had higher levels of flu activity and therefore were more severe flu seasons. The larger proportion of individuals with a health-score decline of >50% in 2012–13 and 2014–15 also suggests these flu seasons may have been more severe.

Despite this possible variation in flu activity levels, when examining the data at the level of symptom severity, the proportion of individuals with a particular set of symptoms visiting a health service showed only very slight variation across years. For example, the risk of an individual with ILI_Fever_ visiting a health service only varied by approximately 1–2.5% over the four years examined. This suggests that surveillance bodies could use this information in conjunction with information on the number of individuals seeking care in a particular year in order to extrapolate an estimation of community burden.

## Conclusions

This data provided evidence that increasing symptom severity, longer duration of illness and larger percentage decreases in reported health-score were associated with increased odds of visiting a health service, suggesting that traditional surveillance systems are indeed capturing more severe episodes of illness which could result in false estimations of seasonal severity. Our data also suggests that the proportion of individuals visiting a health service remains relatively stable within specific sets of symptoms, such as ILI_Fever,_ across years. This data could be used in combination with data on consultation rates to provide better estimates of community burden. Future work could involve assessing the proportion of individuals testing positive for influenza within different ILI categories. Furthermore, Flusurvey could be used monitor the health-seeking behaviour of particular sets of symptom severity and illness duration over additional seasons in order to establish their relative use in providing information on community burden and seasonal severity.

### Additional files

Additional file 1: Figure S1. Correlation between severity indicators. (DOC 93 kb)

Additional file 2: Table S1A. Proportion of illness episodes during which a healthcare service is visited, by severity indicator. (DOCX 13 kb)

Additional file 3: Table S1B. Proportion of illness episodes during which a healthcare service is contacted, by severity indicator. (DOCX 13 kb)

Additional file 4: Figure S2. Seasonal trends in ILI consultation rates and episodes of illness. (DOCX 147 kb)

Additional file 5: Table S2. Crude and adjusted ORs and adjusted LRTs for contacting a health service. (DOCX 16 kb)

Additional file 6: Table S3. Crude ORs for visiting or contacting a health service, excluding 2011–2012. (DOCX 17 kb)

Additional file 7: Table S4A. Odds of visiting a health service, by season. (DOCX 12 kb)

Additional file 8: Table S4B. Odds of contacting a health service, by season. (DOCX 12 kb)

## Appendix 1

### Statistical analyses

Standard errors were monitored to ensure multicollinearity between severity indicators did not make the model unstable. Confounders were then removed one at a time to evaluate their effect on the severity indicators. They were only removed permanently if they were not associated with the outcomes (*p* > 0.1) *and* if their removal increased the difference between the odds ratio (OR) of the severity indicators in univariate models and the OR of the severity indicators in the full models.

Likelihood ratio tests (LRT), which measure goodness of fit, were carried out for univariate and multivariate analyses in order to examine the association of each variable with visiting or contacting a health service (H_0_: No association). To establish whether the odds of health-seeking behaviour varied due to clustering by individual, the LRT for clustering was noted from the fully-adjusted models.

LRTs were also carried out to test whether the effect of symptom severity or illness duration was modified by the year of disease (H_0_: No effect modification).

## Abbreviations

ARI: Acute Respiratory Infection
ECDC: European Centre for Disease Prevention and Control
ILI: Influenza-like illness
OR: Odds ratio
PHE: Public Health England
