# Supplementary figures and images for: Disease severity determines health-seeking behaviour amongst individuals with influenza-like illness in an internet-based cohort
Source: BMC Infect Dis. 2017 Mar 31;17:238. doi: 10.1186/s12879-017-2337-5 (PMC5374571; doi:10.1186/s12879-017-2337-5)

**
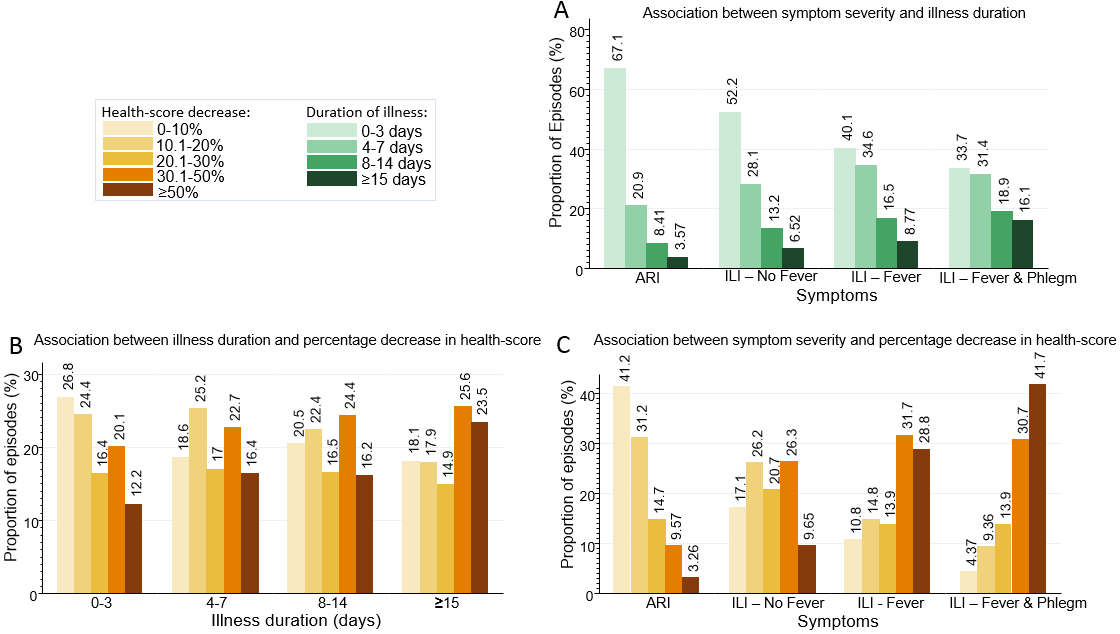
Supplementary Figure 1 – Correlation between severity indicators**

Supplement: Supplementary file 1 — Correlation between severity indicators. (DOC 93 kb) [file 12879_2017_2337_MOESM1_ESM.doc]
