# Supplementary material for: Disease severity determines health-seeking behaviour amongst individuals with influenza-like illness in an internet-based cohort
Source: BMC Infect Dis. 2017 Mar 31;17:238. doi: 10.1186/s12879-017-2337-5 (PMC5374571; doi:10.1186/s12879-017-2337-5)
Supplement: Supplementary file 2 — Proportion of illness episodes during which a healthcare service is visited, by severity indicator. (DOCX 13 kb) [file 12879_2017_2337_MOESM2_ESM.docx]

**Supplementary Table 1A - Proportion of illness episodes during which a healthcare service is visited, by severity indicator**

| **Percentage of illness episodes in each category during which a health service is visited, by season (No.)** | | | | | |
| --- | --- | --- | --- | --- | --- |
|  | **2011-12** | **2012-13** | **2013-14** | **2014-15** | **Combined** |
| **Symptoms** |  |  |  |  |  |
| ARI | 3.8 (22/586) | 4.1 (39/951) | 3.2 (34/1,049) | 4 (35/887) | 3.7 (130/3,473) |
| ILI-No fever | 9 (39/432) | 7.9 (73/925) | 7.5 (69/926) | 10.3 (89/863) | 8.6 (270/3,146) |
| ILI-Fever | 15.4 (27/175) | 16.4 (102/623) | 14.5 (51/352) | 17 (96/564) | 16.1 (276/1,714) |
| ILI-Fever with Phlegm | 29.4 (32/109) | 30.1 (135/448) | 30.4 (66/217) | 32 (116/363) | 30.7 (349/1,137) |
| ARI or ILI | 9.2 (120/1,302) | 11.8 (349/2,947) | 8.7 (220/2,544) | 12.6 (336/2,677) | 10.8 (1,025/9,470) |
| **Illness duration (days)** |  |  |  |  |  |
| 0-3 | 6.2 (45/732) | 6.8 (102/1,501) | 5.4 (78/1,443) | 7.6 (103/1,364) | 6.5 (328/5,040) |
| 4-7 | 11.2 (39/349) | 14.0 (117/834) | 10.0 (62/622) | 13.7 (103/752) | 12.6 (321/2,557) |
| 8-14 | 13.0 (20/154) | 16.9 (65/384) | 16.8 (54/322) | 19.2 (66/343) | 17.04 (205/1,203) |
| ≥15 | 23.9 (16/67) | 28.5 (65/228) | 16.0 (24/150) | 29.5 (64/217) | 25.5 (169/662) |
| **Health-score decrease (%)** |  |  |  |  |  |
| 0-10% | - | 4.3 (20/463) | 2.6 (15/573) | 5.8 (31/536) | 4.2 (66/1,572) |
| 10.1-20% | - | 6.4 (30/468) | 5.4 (29/535) | 5.3 (33/618) | 5.7 (92/1,621) |
| 20.1-30% | - | 8.4 (29/347) | 6.2 (24/388) | 11.8 (45/382) | 8.8 (98/1,117) |
| 30.1-50% | - | 16.2 (78/483) | 15.3 (71/463) | 13.5 (71/527) | 14.9 (220/1,473) |
| ≥50.1% | - | 23.7 (93/392) | 23.0 (51/222) | 32.4 (122/377) | 26.8 (266/991) |
|  |  |  |  |  |  |
