# Supplementary material for: Disease severity determines health-seeking behaviour amongst individuals with influenza-like illness in an internet-based cohort
Source: BMC Infect Dis. 2017 Mar 31;17:238. doi: 10.1186/s12879-017-2337-5 (PMC5374571; doi:10.1186/s12879-017-2337-5)
Supplement: Supplementary file 3 — Proportion of illness episodes during which a healthcare service is contacted, by severity indicator. (DOCX 13 kb) [file 12879_2017_2337_MOESM3_ESM.docx]

**Supplementary Table 1B - Proportion of illness episodes during which a healthcare service is contacted, by severity indicator**

|  | **Percentage of individuals in each category contacting a health service by season (No.)** | | | | |
| --- | --- | --- | --- | --- | --- |
|  | **2011-12** | **2012-13** | **2013-14** | **2014-15** | **Combined** |
| **Symptoms** |  |  |  |  |  |
| ARI | 3.8 (22/586) | 2.8 (27/951) | 2.7 (28/1,049) | 2.6 (23/887) | 2.9 (100/3,473) |
| ILI-No fever | 4.2 (18/432) | 5.6 (52/925) | 4.5 (42/926) | 7.2 (62/863) | 5.5 (174/3,146) |
| ILI-Fever | 10.9 (19/175) | 16.2 (101/623) | 10.2 (36/352) | 13.8 (78/564) | 13.7 (234/1,714) |
| ILI-Fever with Phlegm | 22.9 (25/109) | 22.5 (101/448) | 18.4 (40/217) | 21.2 (77/363) | 21.4 (243/1,137) |
| ARI or ILI | 6.5 (84/1,302) | 9.5 (281/2,947) | 5.7 (146/2,544) | 9 (240/2,677) | 7.9 (751/9,470) |
| **Illness duration (days)** |  |  |  |  |  |
| 0-3 | 4.4 (32/732) | 6.0 (90/1,501) | 3.3 (48/1,443) | 6.0 (82/1,364) | 5.0 (252/5,040) |
| 4-7 | 7.5 (26/349) | 13.0 (108/834) | 6.3 (39/622) | 8.8 (66/752) | 9.4 (239/2,557) |
| 8-14 | 8.4 (13/154) | 11.2 (43/384) | 11.5 (37/322) | 13.7 (47/343) | 11.6 (140/1,203) |
| ≥15 | 19.4 (13/67) | 17.5 (40/228) | 13.3 (20/150) | 20.7 (45/217) | 17.8 (118/662) |
| **Health-score decrease (%)** |  |  |  |  |  |
| 0-10% | - | 3.2 (15/463) | 1.4 (8/573) | 4.5 (24/536) | 3.0 (47/1,572) |
| 10.1-20% | - | 4.3 (20/468) | 3.6 (19/535) | 4.2 (26/618) | 4.0 (65/1,621) |
| 20.1-30% | - | 6.9 (24/347) | 2.8 (11/388) | 7.9 (30/382) | 5.8 (65/1,117) |
| 30.1-50% | - | 11.6 (56/483) | 9.5 (44/463) | 9.1 (48/527) | 10.1 (148/1,473) |
| ≥50.1% | - | 20.4 (80/392) | 17.6 (39/222) | 21.8 (82/377) | 20.3 (201/991) |
