# Supplementary material for: Disease severity determines health-seeking behaviour amongst individuals with influenza-like illness in an internet-based cohort
Source: BMC Infect Dis. 2017 Mar 31;17:238. doi: 10.1186/s12879-017-2337-5 (PMC5374571; doi:10.1186/s12879-017-2337-5)
Supplement: Supplementary file 4 — Seasonal trends in ILI consultation rates and episodes of illness. (DOCX 147 kb) [file 12879_2017_2337_MOESM4_ESM.docx]

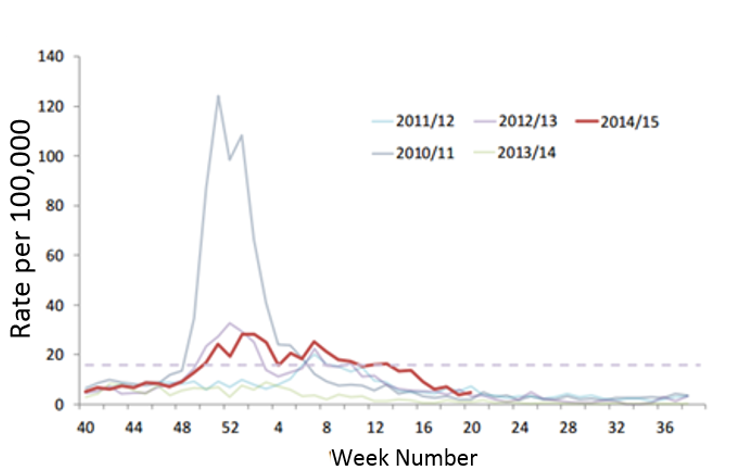

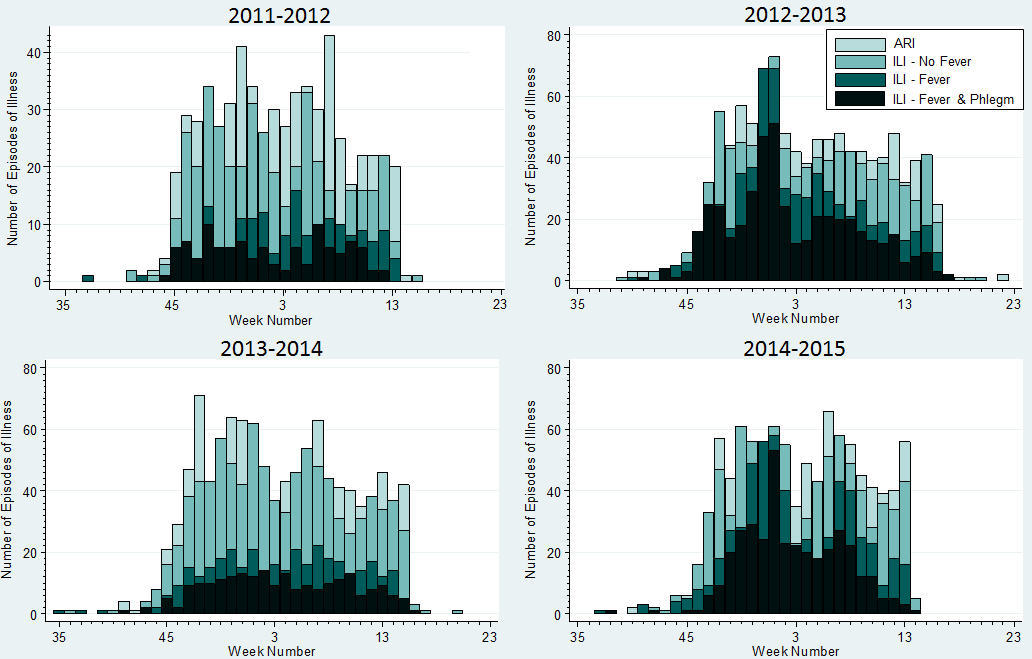
**Supplementary Figure 2 - Seasonal trends in ILI consultation rates and episodes of illness**

**E**

**D**

**C**

**B**

**A**

Images A-D are epidemic curves from Flusurvey data showing episodes of illness. Image E shows General Practice consultation rates for ILI (adapted from PHE, 2015).
