# Supplementary material for: Disease severity determines health-seeking behaviour amongst individuals with influenza-like illness in an internet-based cohort
Source: BMC Infect Dis. 2017 Mar 31;17:238. doi: 10.1186/s12879-017-2337-5 (PMC5374571; doi:10.1186/s12879-017-2337-5)
Supplement: Supplementary file 5 — Crude and adjusted ORs and adjusted LRTs for contacting a health service. (DOCX 16 kb) [file 12879_2017_2337_MOESM5_ESM.docx]

| **Variable** | **Crude ORs (95% CI)*** | **Fully-adjusted ORs (95% CI)** | **LRT p-value for fully-adjusted model** |
| --- | --- | --- | --- |
| **Symptoms** |  |  |  |
| ARI | 1 | 1 | <0.0001 |
| ILI-No Fever | 2.07 (1.58-2.71) | 1.43 (0.99-2.06) |  |
| ILI-Fever | 6.32 (4.73-8.43) | 2.30 (1.52-3.45) |  |
| ILI-Fever with Phlegm | 11.88 (8.63-16.36) | 3.02 (1.94-4.71) |  |
| **Duration (days)** |  |  |  |
| 0-3 | 1 | 1 | <0.0001 |
| 4-7 | 2.10 (1.70-2.60) | 1.65 (1.25-2.17) |  |
| 8-14 | 2.79 (2.16-3.61) | 2.14 (1.53-3.01) |  |
| ≥15 | 5.02 (3.72-6.78) | 2.37 (1.60-3.50) |  |
| **Health-score decrease (%)** |  |  |  |
| 0-10 | 1 | 1 | <0.0001 |
| 10.1-20 | 1.38 (0.92-2.08) | 1.48 (0.95-2.32) |  |
| 20.1-30 | 2.13 (1.41-3.24) | 1.89 (1.19-3.02) |  |
| 30.1-50  % | 4.08 (2.79-5.96) | 2.46 (1.60-3.79) |  |
| ≥50.1 | 10.81 (7.05-16.57) | 4.15 (2.61-6.59) |  |
| **Influenza circulating** |  |  |  |
| No | 1 | 1 | 0.0927 |
| Yes | 1.11 (0.92-1.35) | 0.78 (0.58-1.04) |  |
| **Gender** |  |  |  |
| Male | 1 | 1 | 0.0454 |
| Female | 1.43 (1.18-1.74) | 1.30 (1.00-1.69) |  |
| **Age (years)** |  |  |  |
| 0-18 | 1 | 1 | 0.5668 |
| 19-45 | 0.80 (0.55-1.15) | 1.08 (0.48-2.47) |  |
| 46-65 | 0.83 (0.57-1.19) | 0.95 (0.41-2.17) |  |
| ≥66 | 0.79 (0.51-1.21) | 1.21 (0.50-2.94) |  |
| **Highest qualification** |  |  |  |
| None | 1 | 1 | 0.0926 |
| GCSEs/equivalent | 1.14 (0.70-1.86) | 1.01 (0.50-2.04) |  |
| A-Levels/equivalent | 1.08 (0.69-1.71) | 1.00 (0.51-1.98) |  |
| Undergraduate | 0.77 (0.50-1.20) | 0.65 (0.33-1.29) |  |
| Post-graduate | 0.86 (0.56-1.32) | 0.85 (0.43-1.65) |  |
| **Transport used** |  |  |  |
| Walk/Bike | 1 | 1 | 0.1577 |
| Personal transport | 1.26 (1.00-1.59) | 1.09 (0.79-1.49) |  |
| Public transport | 0.89 (0.68-1.16) | 0.84 (0.58-1.22) |  |
| Other | 4.77 (1.60-14.23) | 4.27 (0.69-26.56) |  |
| **Children in household** |  |  |  |
| No | 1 | 1 | 0.3291 |
| Yes | 1.09 (0.91-1.32) | 0.88 (0.67-1.14) |  |
| **Smoking status** |  |  |  |
| No | 1 | 1 | 0.8045 |
| Yes | 1.10 (0.81-1.50) | 0.95 (0.65-1.40) |  |
| **Flu vaccine** |  |  |  |
| No | 1 | 1 | 0.9315 |
| Yes | 0.89 (0.74-1.07) | 0.99 (0.75-1.30) |  |
| **Asthma** |  |  |  |
| No | 1 | 1 | 0.1781 |
| Yes | 1.19 (0.91-1.55) | 0.77 (0.52-1.14) |  |
| **Allergies** |  |  |  |
| No | 1 | 1 | 0.1102 |
| Yes | 1.21 (1.01-1.44) | 1.22 (0.96-1.55) |  |
| **Diabetes** |  |  |  |
| No | 1 | 1 | 0.9256 |
| Yes | 1.14 (0.71-1.84) | 1.03 (0.54-1.97) |  |
| **Chronic Lung Disease** |  |  |  |
| No | 1 | 1 | 0.0348 |
| Yes | 2.80 (1.55-5.06) | 2.34 (1.10-4.99) |  |
| **Heart Disease** |  |  |  |
| No | 1 | 1 | 0.5458 |
| Yes | 1.24 (0.82-1.88) | 1.19 (0.68-2.07) |  |
| **Renal Disease** |  |  |  |
| No | 1 | 1 | 0.5181 |
| Yes | 1.26 (0.36-4.45) | 0.55 (0.08-3.67) |  |
| **Immunodeficiency** |  |  |  |
| No | 1 | 1 | 0.9532 |
| Yes | 1.47 (0.78-2.77) | 1.03 (0.45-2.35) |  |
| **Self-diagnosis** |  |  |  |
| Cold | 1 | 1 | <0.0001 |
| Flu | 9.42 (7.28-12.20) | 3.43 (2.44-4.83) |  |
| **Flu Season (year)** |  |  |  |
| 2011-12 | 0.61 (0.46-0.82) | - | 0.0730 |
| 2012-13 | 1 | 1 |  |
| 2013-14 | 0.53 (0.42-0.68) | 0.72 (0.52-0.99) |  |
| 2014-15 | 0.93 (0.75-1.15) | 1.00 (0.77-1.30) |  |

**Supplementary Table 2 – Crude* and adjusted ORs and adjusted LRTs for contacting a health service**

* Included all 4 years so as to show the ORs for all 4 seasons. Univariate analyses excluding 2011-12 showed similar results **(Supplementary Table 3)**. Due to the inclusion of health-score, the fully –adjusted model excludes 2011-12.
