# Supplementary material for: Disease severity determines health-seeking behaviour amongst individuals with influenza-like illness in an internet-based cohort
Source: BMC Infect Dis. 2017 Mar 31;17:238. doi: 10.1186/s12879-017-2337-5 (PMC5374571; doi:10.1186/s12879-017-2337-5)
Supplement: Supplementary file 6 — Crude ORs for visiting or contacting a health service, excluding 2011–2012. (DOCX 17 kb) [file 12879_2017_2337_MOESM6_ESM.docx]

**Supplementary Table 3 – Crude ORs for visiting or contacting a health service, excluding 2011-2012 (see page below)**

| Variable | *Crude ORs for visiting a health service (95% CI) | *Crude ORs for contacting a health service (95% CI) |
| --- | --- | --- |
| Symptoms |  |  |
| ARI | 1 | 1 |
| ILI-No Fever | 2.70 (2.06-3.54) | 2.33 (1.72-3.14) |
| ILI-Fever | 6.52 (4.83-8.79) | 7.17 (5.18-9.92) |
| ILI-Fever with Phlegm | 18.43 (13.05-26.03) | 12.99 (9.09-18.57) |
| Duration (days) |  |  |
| 0-3 | 1 | 1 |
| 4-7 | 2.41 (1.94-3.00) | 2.16 (1.72-2.72) |
| 8-14 | 3.86 (2.96-5.02) | 2.92 (2.21-3.85) |
| ≥15 | 7.27 (5.29-9.99) | 4.98 (3.59-6.91) |
| Health-score decrease (%) |  |  |
| 0-10 | 1 | 1 |
| 10.1-20 | 1.46 (1.00-2.11) | 1.38 (0.92-2.08) |
| 20.1-30 | 2.58 (1.76-3.79) | 2.13 (1.41-3.24) |
| 30.1-50 | 5.32 (3.70-7.65) | 4.08 (2.79-5.96) |
| ≥50.1 | 14.38 (9.45-21.88) | 10.81 (7.05-16.57) |
| Influenza circulating |  |  |
| No | 1 | 1 |
| Yes | 1.02 (0.85-1.23) | 1.06 (0.86-1.30) |
| Gender |  |  |
| Male | 1 | 1 |
| Female | 1.51 (1.24-1.84) | 1.52 (1.23-1.88) |
| Age (years) |  |  |
| 0-18 | 1 | 1 |
| 19-45 | 0.44 (0.31-0.63) | 0.74 (0.50-1.10) |
| 46-65 | 0.66 (0.47-0.94) | 0.87 (0.59-1.29) |
| ≥66 | 0.65 (0.43-0.97) | 0.75 (0.47-1.20) |
| Education |  |  |
| None | 1 | 1 |
| GCSEs/equivalent | 1.02 (0.64-1.64) | 1.27 (0.75-2.14) |
| A-Levels/equivalent | 0.99 (0.64-1.54) | 1.25 (0.77-2.04) |
| Undergraduate | 0.67 (0.44-1.02) | 0.84 (0.52-1.34) |
| Post-graduate | 0.61 (0.40-0.92) | 0.90 (0.57-1.42) |
| Transport used |  |  |
| Walk/Bike | 1 | 1 |
| Personal transport | 1.67 (1.30-2.13) | 1.32 (1.02-1.70) |
| Public transport | 0.98 (0.73-1.30) | 0.87 (0.64-1.18) |
| Other | 6.28 (1.62-24.35) | 7.41 (2.02-27.23) |
| Children in household |  |  |
| No | 1 | 1 |
| Yes | 1.18 (0.97-1.42) | 1.12 (0.92-1.37) |
| Smoking status |  |  |
| No | 1 | 1 |
| Yes | 1.43 (1.06-1.94) | 1.21 (0.87-1.68) |
| Flu vaccine |  |  |
| No | 1 | 1 |
| Yes | 1.13 (0.94-1.35) | 0.87 (0.71-1.07) |
| Asthma |  |  |
| No | 1 | 1 |
| Yes | 2.07 (1.61-2.67) | 1.17 (0.87-1.56) |
| Allergies |  |  |
| No | 1 | 1 |
| Yes | 1.27 (1.06-1.52) | 1.24 (1.02-1.50) |
| Diabetes |  |  |
| No | 1 | 1 |
| Yes | 2.34 (1.53-3.58) | 1.21 (0.74-2.00) |
| Chronic Lung Disease |  |  |
| No | 1 | 1 |
| Yes | 3.11 (1.67-5.80) | 3.07 (1.65-5.71) |
| Heart Disease |  |  |
| No | 1 | 1 |
| Yes | 1.51 (0.99-2.29) | 1.22 (0.77-1.93) |
| Renal Disease |  |  |
| No | 1 | 1 |
| Yes | 1.65 (0.45-5.99) | 1.07 (0.25-4.70) |
| Immunocompromised |  |  |
| No | 1 | 1 |
| Yes | 2.58 (1.42-4.69) | 1.41 (0.70-2.81) |
| Self-diagnosis |  |  |
| Cold | 1 | 1 |
| Flu | 8.64 (6.91-10.81) | 9.76 (7.33-12.99) |
| Flu Season (year) |  |  |
| 2012-2013 | 1 | 1 |
| 2013-2014 | 0.65 (0.52-0.82) | 0.52 (0.41-0.67) |
| 2014-2015 | 1.09 (0.89-1.34) | 0.93 (0.75-1.15) |
