# Supplementary material for: Disease severity determines health-seeking behaviour amongst individuals with influenza-like illness in an internet-based cohort
Source: BMC Infect Dis. 2017 Mar 31;17:238. doi: 10.1186/s12879-017-2337-5 (PMC5374571; doi:10.1186/s12879-017-2337-5)
Supplement: Supplementary file 7 — Odds of visiting a health service, by season. (DOCX 12 kb) [file 12879_2017_2337_MOESM7_ESM.docx]

**Supplementary Table 4A – Odds of visiting a health service, by season**

| Fully-adjusted Odds Ratios for visiting a health service (by individual year) | | | | |
| --- | --- | --- | --- | --- |
| ^Symptoms^ | **2011-2012** | **2012-2013** | **2013-2014** | **2014-2015** |
| ^ARI^ | 1 | 1 | 1 | 1 |
| ^ILI-No Fever^ | 3.17 (1.33-7.58) | 1.07 (0.56-2.06) | 1.85 (0.97-3.54) | 1.71 (0.96-3.05) |
| ^ILI-Fever^ | 4.30 (1.55-11.92) | 2.46 (1.18-5.11) | 3.42 (1.56-7.49) | 1.86 (0.98-3.56) |
| ^ILI-Fever with Phlegm^ | 9.77 (2.87-33.25) | 5.98 (2.56-13.96) | 10.78 (4.14-28.09) | 3.92 (1.92-8.01) |
| ^Duration^ |  |  |  |  |
| ^0-3^ | 1 | 1 | 1 | 1 |
| ^4-7^ | 2.48 (1.19-5.15) | 2.42 (1.45-4.04) | 1.99 (1.12-3.51) | 2.15 (1.37-3.39) |
| ^8-14^ | 2.94 (1.22-7.07) | 3.61 (1.88-6.91) | 6.37 (3.09-13.12) | 3.48 (2.01-6.04) |
| ^15+^ | 5.43 (1.63-18.07) | 3.89 (1.87-8.10) | 3.97 (1.70-9.29) | 5.16 (2.67-9.97) |
| ^Health-score^ |  |  |  |  |
| ^0-10%^ | - | 1 | 1 | 1 |
| ^10.1-20%^ | - | 1.60 (0.71-3.60) | 2.71 (1.13-6.47) | 1.18 (0.59-2.34) |
| ^20.1-30%^ | - | 2.36 (1.03-5.42) | 2.32 (0.91-5.92) | 2.88 (1.40-5.92) |
| ^30.1-50%^ | - | 3.50 (1.60-7.65) | 7.32 (2.98-18.01) | 2.65 (1.33-5.27) |
| ^≥50.1%+^ | - | 3.59 (1.58-8.17) | 7.80 (2.88-21.14) | 6.40 (2.96-13.86) |
