# Supplementary material for: Disease severity determines health-seeking behaviour amongst individuals with influenza-like illness in an internet-based cohort
Source: BMC Infect Dis. 2017 Mar 31;17:238. doi: 10.1186/s12879-017-2337-5 (PMC5374571; doi:10.1186/s12879-017-2337-5)
Supplement: Supplementary file 8 — Odds of contacting a health service, by season. (DOCX 12 kb) [file 12879_2017_2337_MOESM8_ESM.docx]

**Supplementary Table 4B – Odds of contacting a health service, by season**

| Fully-adjusted Odds Ratios for contacting a health service (by individual year) | | | | |
| --- | --- | --- | --- | --- |
| ^Symptoms^ | **2011-2012** | **2012-2013** | **2013-2014** | **2014-2015** |
| ^ARI^ | 1 | 1 | 1 | 1 |
| ^ILI-No Fever^ | 1.10 (0.53-2.30) | 1.29 (0.56-2.93) | 0.98 (0.54-1.78) | 1.96 (1.08-3.57) |
| ^ILI-Fever^ | 2.28 (1.04-4.98) | 3.61 (1.40-9.30) | 1.54 (0.78-3.05) | 2.21 (1.14-4.31) |
| ^ILI-Fever with Phlegm^ | 3.75 (1.62-8.68) | 5.47 (1.89-15.85) | 2.09 (1.00-4.40) | 3.09 (1.52-6.30) |
| ^Duration^ |  |  |  |  |
| ^0-3^ | 1 | 1 | 1 | 1 |
| ^4-7^ | 2.09 (1.13-3.88) | 2.61 (1.39-4.92) | 2.01 (1.20-3.37) | 1.07 (0.70-1.64) |
| ^8-14^ | 2.05 (0.95-4.45) | 1.48 (0.69-3.18) | 4.11 (2.34-7.21) | 1.74 (1.06-2.87) |
| ^15+^ | 3.83 (1.61-9.12) | 1.42 (0.58-3.46) | 3.80 (1.91-7.55) | 2.47 (1.40-4.35) |
| ^Health-score^ |  |  |  |  |
| ^0-10%^ | - | 1 | 1 | 1 |
| ^10.1-20%^ | - | 1.25 (0.46-3.41) | 3.18 (1.32-7.64) | 1.06 (0.54-2.06) |
| ^20.1-30%^ | - | 2.12 (0.75-6.00) | 1.96 (0.74-5.19) | 1.66 (0.85-3.27) |
| ^30.1-50%^ | - | 2.10 (0.80-5.49) | 6.14 (2.63-14.33) | 1.57 (0.83-3.00) |
| ^≥50%^ | - | 2.96 (1.05-8.35) | 10.01 (4.14-24.17) | 2.95 (1.51-5.74) |
